# Distress Detection in Subway Tunnel Images via Data Augmentation Based on Selective Image Cropping and Patching

**DOI:** 10.3390/s22228932

**Published:** 2022-11-18

**Authors:** Keisuke Maeda, Saya Takada, Tomoki Haruyama, Ren Togo, Takahiro Ogawa, Miki Haseyama

**Affiliations:** 1 Faculty of Information Science and Technology, Hokkaido University, N-14, W-9, Kita-ku, Sapporo 060-0814, Hokkaido, Japan; 2 Graduate School of Information Science and Technology, Hokkaido University, N-14, W-9, Kita-ku, Sapporo 060-0814, Hokkaido, Japan

**Keywords:** deep learning, distress detection, data augmentation, subway tunnels, maintenance

## Abstract

Distresses, such as cracks, directly reflect the structural integrity of subway tunnels. Therefore, the detection of subway tunnel distress is an essential task in tunnel structure maintenance. This paper presents the performance improvement of deep learning-based distress detection to support the maintenance of subway tunnels through a new data augmentation method, selective image cropping and patching (SICAP). Specifically, we generate effective data for training the distress detection model by focusing on the distressed regions via SICAP. After the data augmentation, we train a distress detection model using the expanded training data. The new image generated based on SICAP does not change the pixel values of the original image. Thus, there is little loss of information, and the generated images are effective in constructing a robust model for various subway tunnel lines. We conducted experiments with some comparative methods. The experimental results show that the detection performance can be improved by our data augmentation.

## 1. Introduction

The subway networks play an essential role in the current urban infrastructure in transporting millions of commuters in major cities [1,2,3]. However, it has been reported in [4] that many concrete structures show significant signs of degradation after only 20 to 30 years due to the joint action of mechanical and environmental effects. Many facilities consisting of concrete structures were intensively built during rapid economic growth, and the number of aging facilities has dramatically increased [5,6]. For instance, cracks, as an important type of distress, can influence the stability of the tunnel structure and require periodic inspection to assess subway line structural integrity and ensure traffic safety. Currently, many countries primarily conduct crack detection in subway tunnels by manual inspection [2]. It has been reported that the annual maintenance costs on a global scale have increased to more than 3% of the world’s gross domestic product [7]. Furthermore, the inspection time outside operating hours of subways is short, from the end of the working day to the beginning of the working day, and there is the possibility of human errors since engineers have been performing their operations based on their knowledge and experience [3,8,9].

Under these circumstances, advanced infrastructure maintenance techniques that can reduce costs and the burden on engineers are needed [10,11,12,13]. Some infrastructure maintenance methods have been proposed based on computer vision with images of infrastructure [14,15,16]. These methods can be roughly divided into the following three categories: image processing-based, machine learning-based, and deep learning-based methods. According to the characteristics of distress, which include color [17], texture [18], and shape [19], image processing-based methods [20], edge detection [21], minimum path [22], and morphology [23] are useful. Although these methods have the advantage of simplicity and practicability because of the strong correlation with specific tasks, the detection accuracy is low for images of distress due to their complex backgrounds. Combing with image processing technology to extract distressed features, machine learning-based methods use support-vector machines [24,25], neural networks [26,27,28,29,30,31], clustering [32], and random forests [27,33,34] to detect distresses. These methods can automatically learn the relationships between distressed features and improve detection performance. However, since the features used by these methods are artificially designed and incomplete, the detection performance is limited by the feature extraction ability. Benefiting from the advances of deep learning technologies in recent years [35,36,37], some methods for detecting distressed regions based on deep learning with images of subway tunnels [38,39,40,41,42,43,44,45] have been proposed. The study [41] proposed a crack detection method based on a deep fully convolutional network (FCN) for the semantic segmentation of concrete crack images. They used concrete crack images collected at various campus buildings of Middle East Technical University [46], but these images were maintained for crack detection, which differs from the characteristics of the data used in actual tunnels. In the method [44], pixel-level crack detection using FCN has also been used to detect cracks in pavement and concrete walls. These studies were conducted on images taken in environments completely different from those of subway tunnels. On the other hand, some methods [45,47] have performed crack detection in subway tunnels, but they focus only on specific lines of tunnels. In the actual situation, there are many subway lines, and the years of operation and operating conditions of each line vary widely. Depending on the subway line to be analyzed, insufficient training data can be collected, or the subway tunnel structure is unsuitable for training the model since the conventional methods are constructed to be specific to a certain subway line. Therefore, it is necessary to construct a model with high generalizability that can consider the diversity of subway lines.

Although several data augmentation methods [48,49,50] have been recently proposed to improve the model’s generalizability, it is essential to select an appropriate data extension method for the training data [51]. Specifically, SamplePairling [49] is a data augmentation method that randomly extracts two images from a set of images and combines them with the same transmittance. Mixup [48] is a data augmentation method that randomly extracts two images from a set of images and combines them with the transmittances determined according to the beta distribution. These methods [48,49] change the original pixel values to augment data. The changes in original pixel values can affect the performance of distress detection. However, random image cropping and patching (RICAP) [50] is a data augmentation method that randomly selects four images from the dataset and combines them to generate a new image. RICAP does not change the original pixel values to augment data. Additionally, RICAP can generate new images containing images of multiple subway lines. In our previous study, we validated the performance of distress detection using each data augmentation method [48,49,50]. The results showed that there is little loss of information using the data augmentation method that does not change the pixel values of the original images, and the generated data are effective for distress detection. In the study [52], data augmentation was conducted by generating new images based on RICAP [50] to include the regions of multiple lines. Since there is little loss of information in the new images generated based on RICAP, the generated images are effective training images for building a robust model for various subway tunnel lines. The detection performance of the distressed regions has been improved using these images as additional training data. However, most images of distress contain regions where no distressed region exists. In this case, the data augmentation methods, such as RICAP, are likely to generate images that contain many regions where no distressed region exists, limiting the improvement of the distress detection performance. Therefore, there is a possibility that we can generate further effective images for building a robust model for various subway tunnel lines by focusing only on the regions where the distressed regions exist.

In this paper, we propose a new data augmentation method, selective image cropping and patching (SICAP), to accurately detect subway tunnel regions of distress by extracting only the distressed regions from the images of distress from multiple lines and combining them to expand the data. The proposed method can improve the performance of distress detection by expanding the data and focusing only on the distressed regions. We can generate a lot of effective data for training the model for distress detection by focusing on the distressed regions via SICAP. After the data augmentation, we construct a distress detection model based on the deep convolutional neural networks from the expanded training data. The new image generated based on SICAP does not change the pixel values of the original image, and there is little loss of information. Additionally, it is an effective training image for building a robust model for various subway tunnel lines since the expanded data invariably contain distressed regions. Therefore, data augmentation based on SICAP can improve the detection of regions of distress in subway tunnels.

For obtaining robustness, recent studies have focused on the pre-training of models based on self-supervised learning (SSL). SSL is an approach for acquiring knowledge from unsupervised data as model parameters and applying them to learning various downstream tasks. While this approach is basically effective, it is necessary to optimize both the training of the SSL model and the model for the downstream task. In research aimed at real-world applications of AI, such as this research, it is undesirable to train multiple models, since the model must be re-trained each time new data are obtained. From the standpoint of such real-world use, data extensions that directly improve the target task are suitable.

The remainder of the paper is organized as follows. In Section 2, we describe our data augmentation method for detecting distressed regions of subway tunnels. Section 3 presents the experimental results to verify the effectiveness of the proposed method. Finally, Section 4 presents the conclusion. For a smooth explanation of the proposed method, the mathematical variables and symbols used in this paper are shown in Table 1.

## 2. Distress Detection Based on Selective Image Cropping and Patching

### 2.1. Selective Image Cropping and Patching

**Figure 1:**
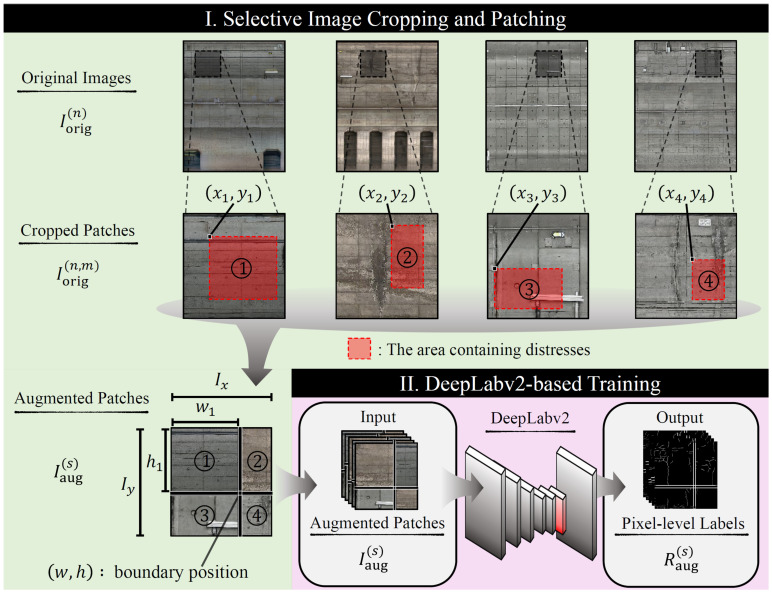
Overview of the proposed method for detecting regions of distress in subway tunnels.

In this section, we explain the proposed data augmentation method, SICAP. Figure 1 shows the overview of the proposed method. The proposed SICAP selects four images from the dataset, performs region extraction for each image, and combines the extracted images to generate a new image. First, we perform patch segmentation on the image of distress $I_{\mathrm{orig}}^{\left(n\right)}$ (n=1,2,⋯,|I|, where I is the set of images of distress $I_{\mathrm{orig}}^{\left(n\right)}$), to obtain the set of patches $I_{\mathrm{orig}}^{\left(n,m\right)}$ ($m=1,2,\cdots ,|I_{\mathrm{orig}}^{\left(n\right)}|$, with $|I_{\mathrm{orig}}^{\left(n,m\right)}|$ being the set of patches to be partitioned from $I_{\mathrm{orig}}^{\left(n\right)}$). Then, we perform data augmentation on patches $I_{\mathrm{orig}}^{\left(n,m\right)}$ by SICAP. Specifically, the proposed method selects four patches $I_{k}$ (k∈{1,2,3,4}) from the set of patches $|I^{\left(n\right)}|$ that contain distress regions and include multiple types of lines. Next, the boundary points (w,h) for generating one image from these regions are calculated according to the beta distribution from the following equations:

(1) $$w=\mathrm{round}\left(w^{\prime }I_{x}\right),\;w^{\prime }∼B\left(\alpha ,\beta \right),$$

(2) $$h=\mathrm{round}\left(h^{\prime }I_{y}\right),\;h^{\prime }∼B\left(\alpha ,\beta \right),$$

where $I_{x}$ and $I_{y}$ are the width and height of the patch $I_{\mathrm{orig}}^{\left(n,m\right)}$. α∈(0,∞) and β∈(0,∞) are hyperparameters, and round(·) is a function that performs fractional processing. The width $w_{k}$ and height $h_{k}$ of each region extracted from the four patches $I_{k}$ are determined using *w* and *h* as follows:

(3) $$\begin{array}{ccc} w_{1} & = & w_{3} \end{array}$$

(4) $$\begin{array}{ccc} & = & w, \end{array}$$

(5) $$\begin{array}{ccc} w_{2} & = & w_{4} \end{array}$$

(6) $$\begin{array}{ccc} & = & I_{x}-w, \end{array}$$

(7) $$\begin{array}{ccc} h_{1} & = & h_{3} \end{array}$$

(8) $$\begin{array}{ccc} & = & h, \end{array}$$

(9) $$\begin{array}{ccc} h_{2} & = & h_{4} \end{array}$$

(10) $$\begin{array}{ccc} & = & I_{y}-h. \end{array}$$

Using $w_{k}$ and $h_{k}$, we determine the coordinates $x_{k}$ and $y_{k}$ for region extraction according to the uniform distribution as follows:

(11) $$x_{k}∼U\left(0,I_{x}-w_{k}\right),$$

(12) $$y_{k}∼U\left(0,I_{y}-h_{k}\right).$$

The determination of $x_{k}$ and $y_{k}$ is repeated until all four regions contain distressed regions. Finally, the four regions extracted based on ($x_{k}$, $y_{k}$) and ($w_{k}$, $h_{k}$) are joined so that they touch at the boundary point (w,h) to generate a new patch $I_{\mathrm{aug}}^{\left(s\right)}$ (s=1,2,⋯,|S|, where S is the set of augmented patches). We also apply the same process into the label $R_{\mathrm{orig}}^{\left(n\right)}$ (n=1,2,⋯,|R|, where R is the set of the distress label $R_{\mathrm{orig}}^{\left(n\right)}$) to obtain a patch $R_{\mathrm{aug}}^{\left(s\right)}$ corresponding to the new patch $I_{\mathrm{aug}}^{\left(s\right)}$. Consequently, the data augmentation using SICAP for the distressed images of subway tunnels can generate the training data for constructing robust models for various subway tunnel lines.

The previous study [50] claims that RICAP replaces the classification task with the occupancy estimation task by mixing the four class labels with ratios proportional to the areas of the four cropped images. They also claim that RICAP forces the CNN to classify each pixel in a weakly supervised manner, and thereby, the CNN can focus on minor features, partial features, backgrounds, and any other information that is often ignored. Therefore, although there may not be a significant change in the diversity of the data, the data augmentation with RICAP and SICAP will allow a variety of objects to be recognized more efficiently than with the original images alone. Furthermore, pixel-wise blending methods such as mixup [48] superimpose images, which can lead to over-focusing on salient regions in the image. On the other hand, RICAP and our SICAP are spatial blending approaches that can effectively learn the original local features and newly extract global features from the combined images. Thus, this approach avoids overfitting compared to conventional data augmentation methods.

### 2.2. Distress Detection with DeepLabv2

In this section, we perform the training of DeepLabv2 [53], which detects the distressed region for each input image pixel, using the patches acquired from the images of distress and the corresponding pixel-level distress labels. DeepLabv2 is a model that introduces an Atrous convolution layer into ResNet-101 [36]. The Atrous convolution layer performs wide-area convolution compared to the normal convolution layer to achieve robust features. We replace the classification layer in ResNet-101 with Atrous spatial pyramid pooling (ASPP). ASPP applies the Atrous convolution layer on multiple scales and integrates and pools features spatially. This approach is inspired by the success of the spatial pyramid pooling method [54], which shows that regions of an arbitrary scale can be accurately and efficiently classified by resampling convolutional features extracted at a single scale. ASPP has been implemented as a variant of the scheme that uses multiple parallel Atrous convolutional layers with different sampling rates. The features extracted for each sampling rate are further processed in separate branches and fused to generate the final result. Therefore, DeepLabv2 can calculate the feature map with an Atrous convolution feature layer while maintaining the resolution.

In the training phase, we take a patch-based training strategy since subway tunnel images have high resolution. In the proposed method, we divide subway tunnel images into multiple patches and perform data augmentation based on SICAP. Then, we use the augmented patches $I_{\mathrm{aug}}^{\left(s\right)}$ to train our network. The network is initialized with parameters trained on the ImageNet dataset [55]. We perform a multi-class classification, such as “cracks”, “all the others”, and “no distress”, to detect the distressed region for each pixel of the input image. Since the pixels of the background class that do not contain a distressed region are more than those of the distress classes, the training may be relatively dominated by the background class. The final classification layer of the DeepLabv2 network is a spatial pyramid pooling layer consisting of several classification layers with different dilation rates. Layers with different dilation rates have different receptive field sizes, thus facilitating multiple-scale detection. At the end of the training, we use validation data to observe the training process and fix the parameters of our model. Note that the data are class-unbalanced due to the overwhelming number of background areas. To consider this situation, we added weights to the losses related to crack segmentation during the DeepLabv2 training.

In the test phase, we divide subway tunnel images into multiple patches, input the test patches into the trained model, and estimate the distressed regions. The results of each test patch are integrated into a single image. In this way, we successfully perform distress detection. In the proposed method, we train the model with patches generated based on SICAP so that the patches invariably contain distressed regions. Thus, we can build a robust model for various subway tunnel lines.

## 3. Experimental Results

### 3.1. Dataset

**Figure 2:**
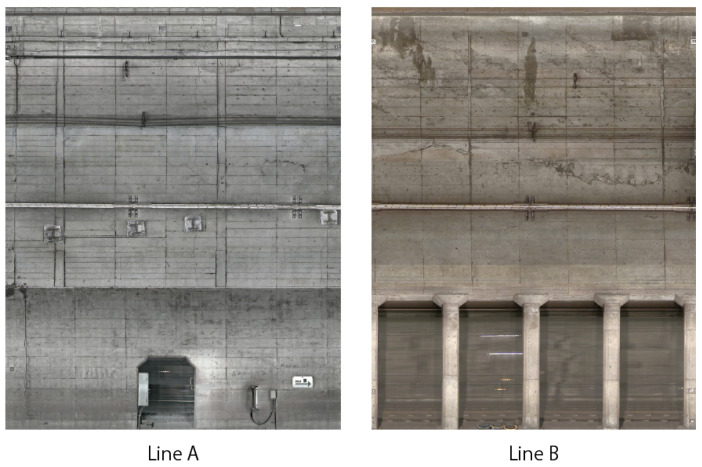
Examples of image of two lines (lines A and B) in the dataset.

**Figure 3:**
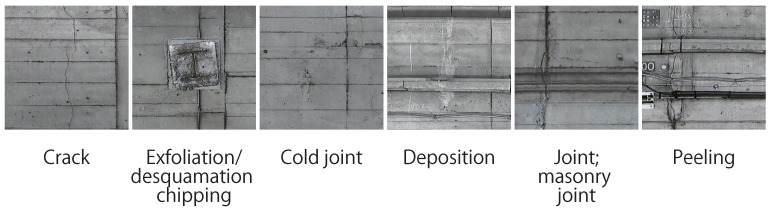
Examples of distresses in the dataset.

**Figure 4:**
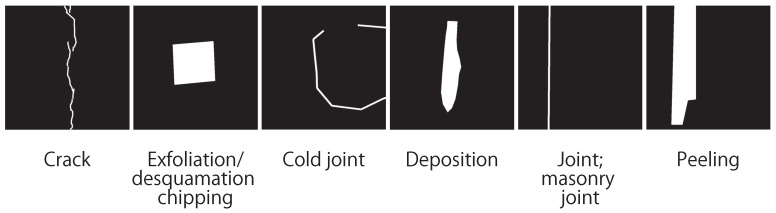
Examples of labels in the dataset, corresponding to Figure 3.

In this section, we explain the dataset used in our experiments. The dataset used in our experiment consists of distressed images of two lines (lines A and B), and their corresponding distress labels were provided by Tokyo Metro Company Limited. Figure 2 shows some examples of images in the dataset. Since the years of operation and the operating conditions of each line vary, the condition of the tunnels and the color of the images vary widely. Specifically, 44 images of distress were taken for each line (lines A and B). The images were taken as RGB images of approximately 5000 × 6400 pixels. We adopted “crack”, “desquamation chipping”, “cold joint”, “deposition”, “masonry joint”, and “peeling”, as shown in Figure 3. In the images of distress, we have pixel-level labels of distress assigned by technical experts, as shown in Figure 4. Each label in Figure 4 corresponds to the kind of distress in Figure 3.

### 3.2. Settings

In this section, we describe our experimental settings. We conducted the experiment and ablation study to validate the effectiveness of the proposed method. In the experiment, we perform distress detection using SICAP for all kinds of images of distress using two subway lines to verify the robustness of the proposed method for various lines. In the ablation study, we perform distress detection using SICAP for each kind of distress using one subway line to verify the effectiveness of selecting the target distress.

We first explain the experimental settings. We selected 44 images from each of lines A and B. We used five images for validation and five images for the test from the dataset. Since the images of distress were taken with high resolutions, we divided them into patches with a patch size of 256 × 256 pixels and a slide width of 100 pixels. We generated 72,238 and 75,240 patches from the images of distress of lines A and B, respectively. The distress labels used in this experiment were “crack” and “all the others”, according to the literature [47]. Note that the “all the other” label includes “desquamation chipping”, “cold joint”, “deposition”, “masonry joint”, and “peeling”. The DeepLabv2 network was trained to classify each pixel in the input image into three classes: “cracks”, “all the others”, and “no distress”. Note that the hyperparameters α and β were set to 5.0. In the training phase, we used stochastic gradient descent as the optimizer with a batch size of 16, momentum of 0.9, and weight decay of $5\times 10^{-4}$, and the number of training epochs was four.

In this experiment, we used intersection over union (IoU) as the evaluation index, which is defined by the following equation:

(13) $$\mathrm{IoU}=\frac{\mathrm{TP}}{\mathrm{TP}+\mathrm{FN}+\mathrm{FP}},$$

where TP, FN, and FP are the number of true-positive, false-negative, and false-positive samples, respectively.

We used the following comparative methods (CMs1–6) in this experiment to verify the effectiveness of the proposed method.

- CM1: A method using patches obtained from images of distress of lines A and B together without data augmentation.
- CM2: A data augmentation method based on SamplePairing [49] for CM1. Two images are randomly extracted from the set of images and combined with the same transmittance.
- CM3: A data augmentation method based on SamplePairing [49] using only patches that contain regions of distress for CM1 (Selective SamplePairing).
- CM4: A data augmentation method based on Mixup [48] for CM1. Two images are randomly extracted from the set of images to be augmented, and their transmittances are determined according to the beta distribution.
- CM5: A data augmentation method based on Mixup [48] using only patches that contain regions of distress for CM1 (Selective Mixup).
- CM6: A data augmentation method based on RICAP [50] for CM1. This corresponds to the conventional method [52] in our research.

Note that CMs2–5 change the original pixel values when performing data augmentation. The reasons for adopting each comparison method are described below. We verified the effectiveness of introducing data augmentation by comparing ours with CM1. CMs 2, 4, and 6 are common data augmentation methods, as described in the introduction, and we verified their effectiveness as data augmentation methods by comparing ours with these methods. In addition, CMs 3 and 5 are data augmentation methods that introduce the supervised information into CMs 2 and 4, respectively. By comparing the proposed method with these methods, we verified the effectiveness of the proposed method as the data augmentation method with the supervised information.

Next, we explain the settings of the ablation study. The number of pixels in the region of distress in the training data varies significantly for each distress. Therefore, it is expected to improve the final detection performance by reducing the influence of the distressed region other than the detection target by performing SICAP for each arbitrary distress. Therefore, in this experiment, we applied SICAP to each arbitrary distress in the detection of subway tunnel images to verify the detection performance. The distress labels used in this experiment were “crack”, “peeling”, and “all the others”. The DeepLabv2 network was trained to classify each pixel in the input image into four classes: “cracks”, “peeling”, “all the others”, and “no distress”. Note that the hyperparameters α and β were set to 5.0; the number of training epochs was 4, and the batch size was 16. In this experiment, we used IoU as the evaluation index, as in the first experiment. We calculated the IoU of each distress and used the average of the IoU when detecting “crack” and “others” as the evaluation index. We used the following comparative methods (CM1–2) in this experiment to verify the effectiveness of the proposed method.

- CM1: A method using patches obtained from images of distress of line A without data augmentation.
- CM2: A data augmentation method based on RICAP [50] for CM1. This corresponds to the conventional method [52] in our research.

### 3.3. Experiment: Performing SICAP for All Kinds of Distress

In this section, we describe the experimental result and quantitative and qualitative evaluation of the proposed method. Table 2 presents the experimental results of IoU for each data augmentation method when the test data are the images of distress of lines A and B. The number of augmented patches of each line is 1.5 times the original patches. As presented in Table 2, the averages of the IoU of the proposed method are higher than those of the other comparative methods in all cases of the test images from lines A and B. The comparison of the IoU between CM1 and PM suggests the effectiveness of performing the data augmentation for the training images. Additionally, the comparison of the IoU among CMs2–6 and PM suggests the effectiveness of SICAP for data augmentation for distress detection. Furthermore, the comparison of the IoU of CM6 and PM suggests the effectiveness of focusing only on the regions where distress exists, especially for RICAP, in the data augmentation method. These results show that data augmentation focusing only on distressed regions without changing the original pixel values is effective in detecting subway tunnel distress.

**Figure 5:**
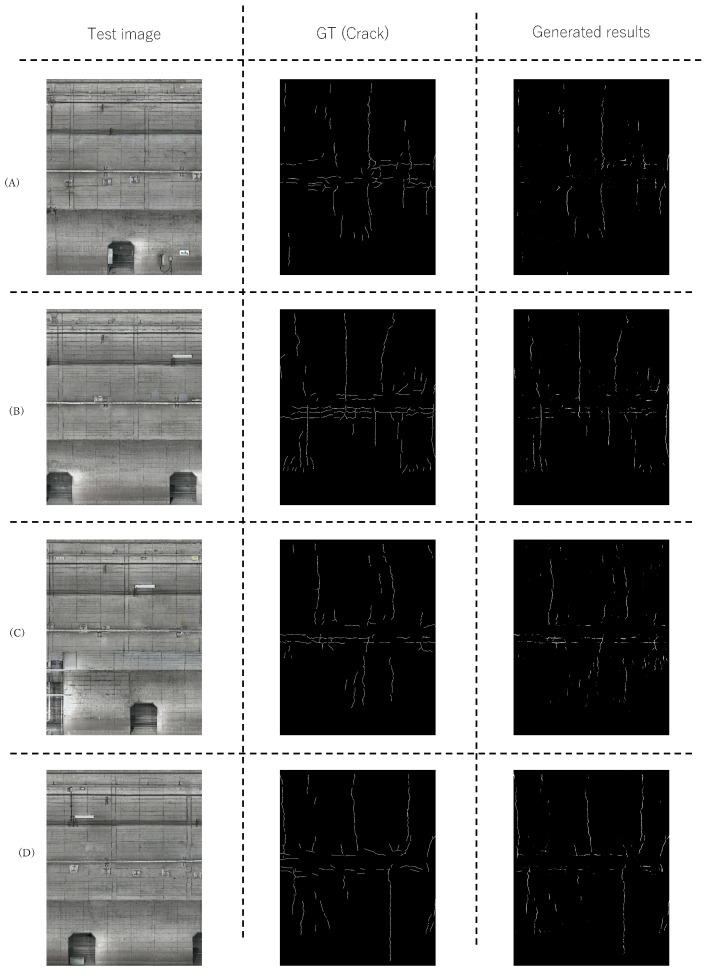
Examples of the resultsof the proposed method in line A. Figures (**A**–**D**) show the tunnel walls at various locations on line A.

**Figure 6:**
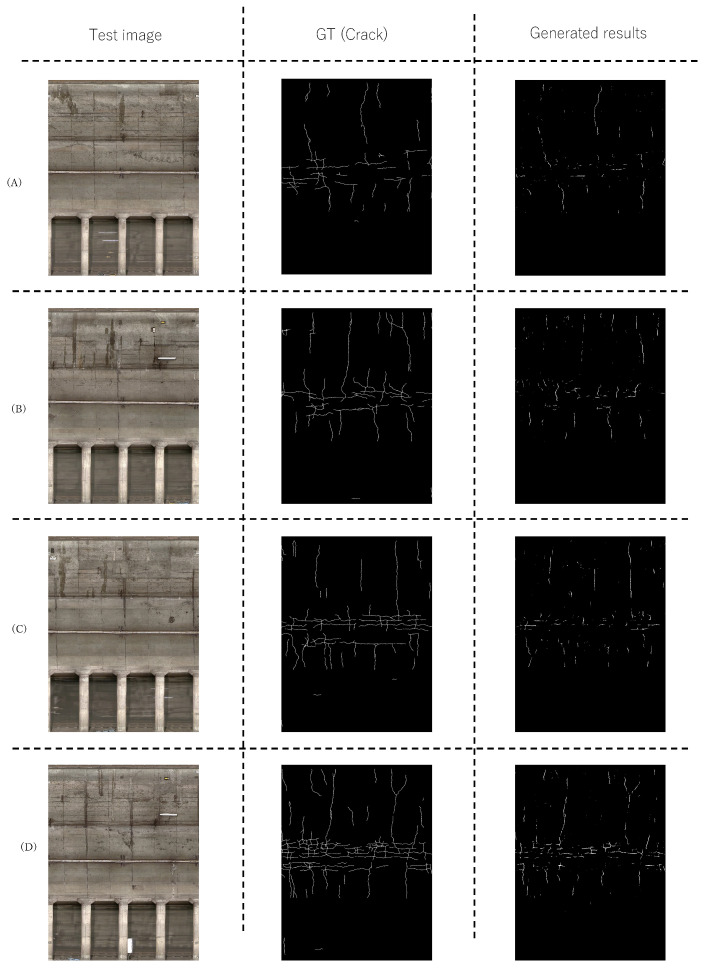
Examples of the results of the proposed method in line B. Figures (**A**–**D**) show the tunnel walls at various locations on line B.

We qualitatively validated the performance of the proposed method. Figure 5 and Figure 6 show the detection results using the proposed method on lines A and B, respectively. The images in the left column are test images used in the experiment. The images in the center column are the pixel-level ground truth corresponding to the test images. The images in the right column are the estimated region using the proposed method. Figure 5 and Figure 6 show that the proposed method can accurately estimate the distressed regions for the most part and can accurately detect a long and bold crack. These results suggest that data augmentation without changing the original pixel values effectively improves the performance of subway tunnel distress detection. Although there are many joints, most of them are not included in the detection results. Rather, the proposed method can detect cracks with high accuracy since it detects horizontal cracks without detecting horizontal joints. The above experimental results confirm the effectiveness of the proposed method.

**Figure 7:**
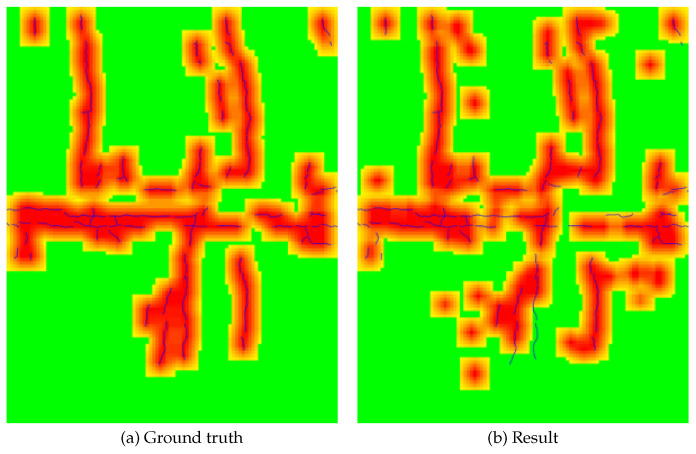
Examples of region-based results. The examples correspond to Figure 5C. Cracks in the image represent the ground truth. The closer the color in a patch is to red, the more positive patches are included.

We demonstrate the effectiveness of the proposed method through a new evaluation that takes into account real-world applications. Specifically, since one of the most important aspects of tunnel maintenance is the density of cracks, a region-based evaluation is performed. For this evaluation, the image is divided into patches of 50 pixels of slide width and 200 × 200 pixels. If cracks are present in 1% or more of the pixels in each patch, the patch is then considered positive; otherwise, it is considered negative. We adopted the f-value to compare the results of our methods with the ground truth. As an example, Figure 7 shows the correct response region in Figure 5C and the region estimated by the proposed method. This region-based comparison confirms that it is possible to qualitatively and adequately identify the cracked area. Using the f-value as the region-based evaluation in this image is about 80%. Therefore, it was confirmed that the performance is absolutely high quantitatively. This method has already reached the level of practical application and is being considered for introduction into the system of a collaborating company.

### 3.4. Ablation Study: Performing SICAP for a Certain Kind of Distress

In this section, we describe the results of the ablation study and quantitative and qualitative evaluation of the proposed method. In this experiment, “PM-Crack” and “PM-Peeling” apply SICAP only to the area of “cracks” and “peeling”, respectively. “PM-Others” applies SICAP only to the area of “others”, including “desquamation chipping”, “cold joint”, “deposition”, and “masonry joint”. In each method, the number of patches used for training is determined experimentally. Table 3 presents the experimental results of the IoU and the number of patches used for training for each method when the test data are the distress images of lines A. As presented in Table 3, the average IoU values of PM are higher than those of the other comparative methods in all cases where the test images are the distress images. Note that the methods showing the highest IoU values for “crack”, “peeling”, and “other distress” are “PM-Crack”, “PM-Peeling”, and “PM-Others”, respectively. Thus, the proposed method effectively improves the detection performance of arbitrary distress in the images of distress in subway tunnels.

**Figure 8:**
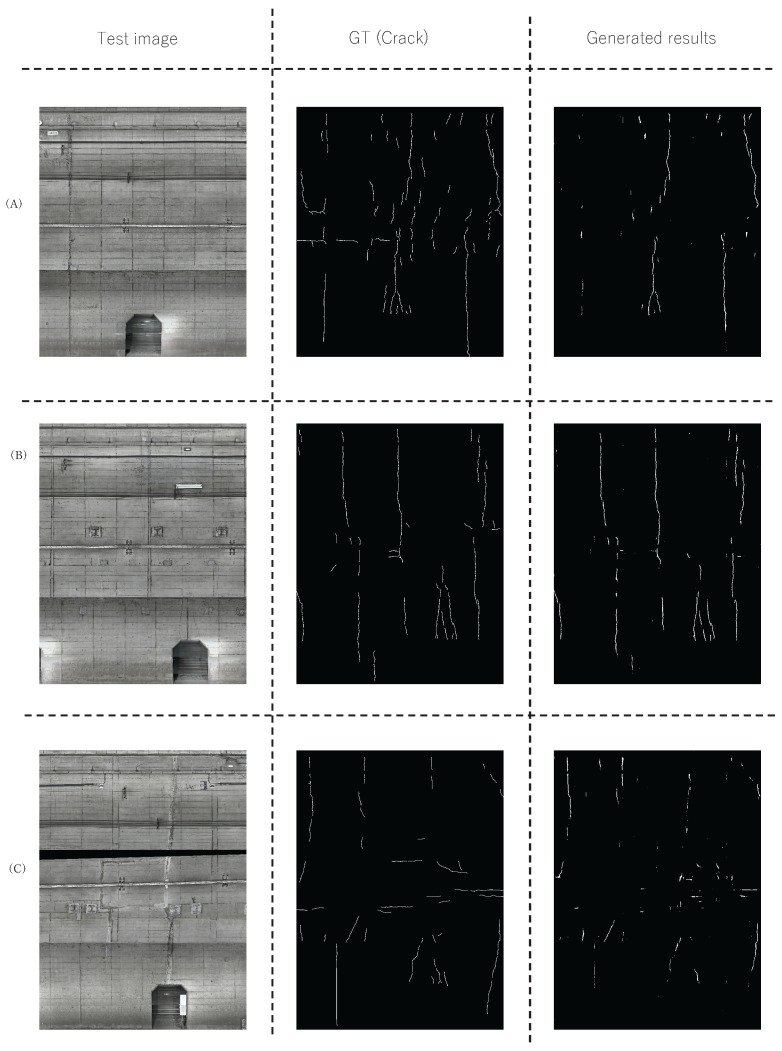
Examples of the results of the proposed method in the ablation study. Figures (**A**–**C**) represent each test image.

Figure 8 shows the detection result using the proposed method performed only for “crack” (PM-Crack) on line A. The images in the left column are test images used in the experiment. The images in the center column are the pixel-level ground truth corresponding to the test images. The images in the right column are the estimated region using the proposed method. Figure 8 shows that the proposed method can accurately estimate the distress regions. Therefore, we improved the final detection performance by reducing the influence of the region of the distress other than the detection target by performing SICAP for each arbitrary distress. The above experimental results confirm the effectiveness of the proposed method when performed for a certain kind of distress.

## 4. Conclusions

In this paper, we proposed a new data augmentation method, SICAP, for distress detection to support the maintenance of subway tunnels. The proposed SICAP extracts only the distress regions from the images of distress of multiple lines and combines them to expand the data. The new image generated based on SICAP does not change the pixel values of the original image; thus, there is little loss of information. Additionally, since the expanded data invariably contain distressed regions, they are effective in building a robust model for various subway tunnel lines. Our new approach quantitatively outperformed the other data augmentation methods. We also confirmed the effectiveness of the proposed method by constructing a distress-specific detection model.

It is necessary to consider the introduction of other subway lines. This will be done as future work, as acquiring a sufficient amount of data is needed. Furthermore, we address the comparison with other data augmentation algorithms as future work.

## Figures and Tables

**Table 1:** Explanation of the mathematical variables and symbols.

Variables	Meanings
*n*	Index of distress images
*m*	Index of patches
$I_{\mathrm{orig}}^{\left(n\right)}$	*n*-th distress image
$I_{\mathrm{orig}}^{\left(n,m\right)}$	*m*-th patch extracted from $I_{\mathrm{orig}}^{\left(n\right)}$
$I_{\mathrm{aug}}^{\left(s\right)}$	*s*-th augmented patches
(*w*, *h*)	Boundary position of the augmented patch $I_{\mathrm{aug}}^{\left(s\right)}$
$w_{k}$, $h_{k}$	Width and height of each region extracted from patch $I_{k}$
$I_{x}$, $I_{y}$	Width and height of patch $I_{\mathrm{orig}}^{\left(n,m\right)}$
$R_{\mathrm{orig}}^{\left(n\right)}$	Ground truth of image $I_{\mathrm{orig}}^{\left(n\right)}$

**Table 2:** Average of IoU when detecting “crack” and “others” based on each data augmentation method (DA method) in experiment I.

	DA Method	Line A			Line B		
		Crack	Others	Average	Crack	Others	Average
CM1	-	0.2600	0.4884	0.3741	0.2278	0.6201	0.4240
CM2	SamplePairing [49]	0.2878	0.3168	0.3023	0.2084	0.5862	0.3973
CM3	Selective SamplePairing	0.2763	0.3872	0.3318	0.1827	0.6471	0.4149
CM4	Mixup [48]	0.2751	0.4443	0.3597	0.2365	0.6073	0.4219
CM5	Selective Mixup	0.2479	0.2514	0.2497	0.1270	0.5254	0.3262
CM6	RICAP [50]	0.2781	0.5177	0.3979	0.2467	0.6229	0.4348
PM	SICAP	0.2983	0.5120	0.4052	0.2171	0.6547	0.4359

**Table 3:** Number of patches used for training after data augmentation and the average of IoU when detecting “crack”, “peeling”, and “others” based on each data augmentation method (DA method) in the ablation study.

	DA Method	Number of Patches				IoU		
		No Distress	Crack	Peeling	Others	Crack	Peeling	Others
CM1	-	75,265	19,304	3555	2483	0.3417	0.1018	0.4332
CM2	RICAP [50]	151,075	51,830	12,800	7730	0.3236	0.1147	0.4096
PM-All	SICAP	151,075	94,899	46,007	33,445	0.3094	0.1355	0.4982
PM-Crack	SICAP	94,569	38,608	3555	2483	0.3503	0.1242	0.4635
PM-Peeling	SICAP	91,014	19,304	19,304	2483	0.3219	0.1583	0.4098
PM-Others	SICAP	92,086	19,304	3555	19,034	0.3355	0.1423	0.5036

## Data Availability

Not applicable.
